# Two-Dimensional CuMn-Layered Double Hydroxides: A Study of Interlayer Anion Variants on the Electrochemical Sensing of Trichlorophenol

**DOI:** 10.1021/acs.inorgchem.3c04568

**Published:** 2024-01-23

**Authors:** Balasubramanian Sriram, Megha Maria Stanley, Sea-Fue Wang, Yung-Fu Hsu, Mary George

**Affiliations:** † Department of Materials and Mineral Resources Engineering, National Taipei University of Technology, Taipei 106, Taiwan; ‡ Department of Chemistry, Stella Maris College, Affiliated to the University of Madras, Chennai, Tamil Nadu 600086, India

## Abstract

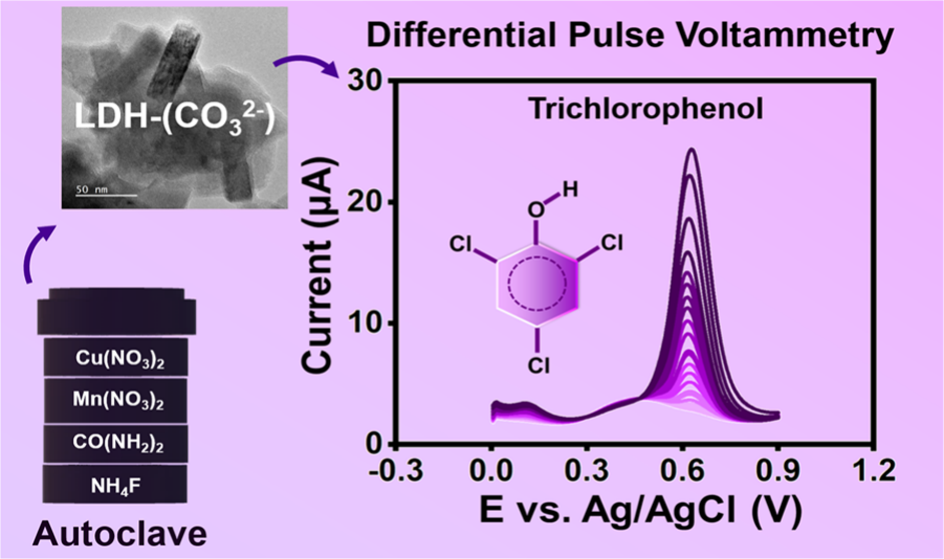

Despite their diverse application profile, aromatic organochlorides such as 2,4,6-trichlorophenol (TP) are widely renowned for creating a negative toll on the balance of the ecosystem. Strict regulatory regimes are required to limit exposure to such organic pollutants. By deployment of a straightforward detection scheme, electrochemical sensing technology offers a competitive edge over the other techniques and practices available for pollutant monitoring. Here, we present a streamlined hydrothermal approach for synthesizing copper–manganese layered double hydroxide (CuMn-LDH) rods to be employed as electrocatalysts for detecting TP in various media. With a focused intention to leverage the full potential of the prepared CuMn-LDHs, the interlamellar region is configured using a series of intercalants. Further, a thorough comparative analysis of their structures, morphologies, and electrochemical performance is accomplished using various analytical techniques. The electrocatalytic oxidation ability of the CuMn-LDH toward TP molecules is markedly altered by incorporating various anions into the gallery region. The dynamic attributes of the developed sensor, such as a wide linear response (0.02–289.2 μM), a low detection limit (0.0026 μM), and good anti-interfering ability, acclaim its superior viability for real-time detection of TP with exceptional tolerance to the presence of foreign moieties. Hence, this work manifests that the nature of intercalants is a vital aspect to consider while designing LDH-based electrochemical probes to detect priority pollutants.

## Abstract 2

This study aims to elucidate the role of the anionic intercalants on the catalytic activity and current response of CuMn-LDHs toward trichlorophenol detection.

## Introduction

As versatile as they are, chlorinated aromatics stealthily harm the environment and risk human life.^1^ For instance, 2,4,6-trichlorophenol (TP) is used extensively as a process intermediate, wood preservative, and germicidal agent and is quite often released into the environment in conjunction with fossil fuel combustion, sludge processing, chlorination, and disinfection of water.^2,3^ With its potency to foster long-term damage to the ecosystem, TP presents a category of high-risk synthetic substances.^4^ Several concerns arise from the high degree of stability of their C–Cl bonds as they inhibit the degradation of TP, leading to bioaccumulation in the environment and the human body.^5^ Particularly, the ease of absorption of TP into the bloodstream allegedly disrupts the normal functioning of endocrine receptors. TP is also known to suppress the action of acetylcholinesterase and thus is widely branded as a potent neurotoxin.^2,6^ In recognition of the toxicological effects such as histopathological concerns, mutagenicity, carcinogenicity, neurotoxicity, and genotoxicity, the US Environmental Protection Agency has classified TP as a priority toxicant.^4,7^ Given the extensive list of adverse health impacts, it is imperative to implement appropriate monitoring strategies for quantifying TP in various media.^8^

The trace-level evaluation of organochloride pollutants in complex environments demands the introduction of affordable, sensitive, and rapid detection technologies.^9^ However, relying on customary chromatographic and spectrophotometric techniques for this purpose falls short of delivering fast, sensitive, and accurate results.^10^ With its simplicity of operation, prompt *in situ* response, high sensitivity, and selectivity, the onset of electrochemical sensing strategies has brought in an evolutionary upgradation in the detection strategies available for target analytes.^3,8,11−13^ The heart of the electrochemical technique lies in its simple electrode preparation, which accommodates a wide range of catalyst materials.^14,15^ Electrocatalyst modification offers the authority to dial in materials that accelerate the ion transfer kinetics for targeted analyte detection.^16^ Several electrocatalysts have been developed to monitor the presence of priority pollutants.^17−19^ For instance, a copper oxide-based electrochemical sensing platform developed by *Jamil A. Buledi* and co-workers successfully recognized trace levels of TP in real-time samples. By incorporating Cu-based nanostructures into the catalyst, the conductive properties of the catalyst are improved, providing a reliable, robust, and affordable sensing platform.^8^ In another effort, Venkatesh and associates developed manganese vanadate nano pebbles to determine TP. The results of the voltammetric analysis revealed that the fabricated sensor demonstrated a wide linear response range and a low detection limit, proving its suitability for real-time sensing.^20^

A wealth of scientific evidence presents LDH-based electrochemical sensors as affordable alternatives to traditional electrocatalysts owing to their remarkable hosting and catalytic capabilities.^21,22^ Typically, LDH embodies a class of anionic clays bearing the formula [M_1–*x*_^2+^M_*x*_^3+^(OH)_2_]^*x*+^[A_*x*/*n*_^*n*–^]^*x*−^. *n*H_2_O.^21^ The LDH frameworks formed by the periodic stacking of positively charged, brucite-like octahedral layers confine charge-compensating anions between the cationic layers to ensure charge neutrality.^23^ While the cationic layer can host various bivalent and trivalent metal cations, the anionic zone can accommodate several organic and inorganic moieties.^24^ A consensus is that trivalent ions replace a fraction of divalent ions from LDH matrix sites.^25^ More importantly, incorporating transition metal-based LDHs is highly beneficial in terms of environmental benignity, abundant availability, and economic viability without compromising the catalytic performance.^26^ Owing to the porous lamellar structure and admirable guest–host synergies, LDH-modified electrodes are presented as versatile electrochemical probes. The rapid diffusion of analytes and abundant active sites contribute to this improved activity. In a related attempt, Asif et al. constructed a CuMn@CNT nanohybrid to precisely quantify H_2_S efflux, wherein the structured integration of nanoarchitectures enabled a highly sensitive pollutant analysis.^27^ Accordingly, the large number of surface active sites and smooth ion transportation ability of CuMn-LDH mark it as an excellent catalyst material that produces an excellent electrochemical performance.

While this is true, the activation energy barrier remains a challenge for optimum sensor performance. The incredible compositional flexibility of LDHs can be leveraged to address this issue. The identity of the divalent and trivalent metal ions and anionic intercalants can be readily exchanged, enabling the synthesis of LDHs with fine-tuned characteristics.^28^ Researchers have acknowledged that, in addition to maintaining the charge neutrality in the LDH structure, the anions (e.g., CO_3_^2–^, Cl^–^, SO_4_^2–^, OH^–^, and NO_3_^–^) present in the gallery region can influence the electrochemical response of the LDH electrodes.^21,29^ The direct coordination of anions with metal centers allows them to modulate the electrical characteristics without reducing the number of active metal centers. For instance, Hunter et al. explored the effect of interlayer anions on the water oxidation activity of NiFe LDHs. The studies showed that incorporating carbonate anions yielded the highest electrochemical activity.^30^ DFT studies by Liu et al. explored the role of guest anions in the construction of LDH electrocatalysts. The results indicate that interactions between anions and the host layer influence the electrochemical performance of LDH.^31^

This study aims to elucidate the role of the anionic intercalants on the catalytic activity and current response of CuMn-LDHs toward TP. It will further pave the way for enhancing the efficiency of LDH-based electrochemical investigations. The rationalized hydrothermal synthesis protocol aids in the development of regulated morphologies. The inclusion of a transition element in a host environment may increase the effectiveness of the electron movement. In addition, given its low cost, availability, and varied valence states, Cu–Mn is well established as an influential agent on catalytic performance. Over the past decades, several anions have been incorporated in the gallery region and reported to alter the electrochemical properties of LDH electrodes in addition to maintaining charge neutrality. However, a detailed comparison of the commonly used anions. In this vein, we exchanged the identities of the anionic intercalants and analyzed the changes in the electrochemical sensing capabilities. As expected, the anions present in the gallery region greatly influenced the electrochemical response of the LDH electrodes. The prepared LDHs are meticulously compared for structural and morphological features, transport kinetics, and electrochemical responsiveness. Further, they are employed as high-efficiency catalysts for the electrochemical measurement of TP. As far as we know, this is the first study to utilize CuMn-LDH as a catalyst for determining TP.

## Materials and Methods

### Chemicals and Reagents

Copper(II) nitrate (Cu(NO_3_)_2_·3H_2_O), Manganese(II) nitrate (Mn(NO_3_)_2_·4H_2_O), urea (CO(NH_2_)_2_), ammonium fluoride (NH_4_F), 2,4,6–Trichlorophenol (TP), [Fe(CN)_6_]^4–,3–^, sodium hydroxide (NaOH), potassium chloride (KCl), sodium phosphate dibasic (Na_2_HPO_4_), sodium dihydrogen phosphate (NaH_2_PO_4_), and ethanol were procured. Ultrapure fresh water is obtained from a Millipore water purification system (Milli-Q, specific resistivity >18 MΩ·cm, S.A.; Molsheim, France) and is used in all the experiments, and Na_2_HPO_4_ and NaH_2_PO_4_ are utilized to prepare 0.1 M (pH 7) PB (phosphate buffer). All of the electrochemical experiments are carried out using 0.1 M PB (pH 7) as the supporting electrolyte.

### Preparation of Carbonate Intercalated CuMn-LDH-(CO_3_^2–^)

For the synthesis of CuMn-LDH–CO_3_^2–^, Cu(NO_3_)_2_·3H_2_O and Mn(NO_3_)_2_·4H_2_O precursors are stoichiometrically (3:1) dissolved in 150 mL of DI water. The homogeneity of the solution is ensured by allowing the solution to be magnetically stirred for 10 min. This is followed by the addition of 1 g of urea and 0.7 g of NH_4_F under stirring conditions. The resultant homogeneous mixture is transferred to a Teflon-lined autoclave and is subjected to hydrothermal treatment at 120 °C for 6 h. The formed precipitates are vacuum-dried at 80 °C for 3 h to remove any trace of undesirable species. The obtained product is termed CuMn-LDH-(CO_3_^2–^).

The possible mechanism for the formation of CuMn-LDH by hydrothermal treatment can be illustrated by the following reactions (eqs 1–5)
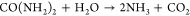
1
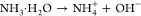
2
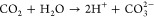
3
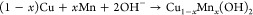
4
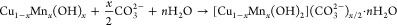
5

### Anion Exchange Treatments

**Scheme 1:**
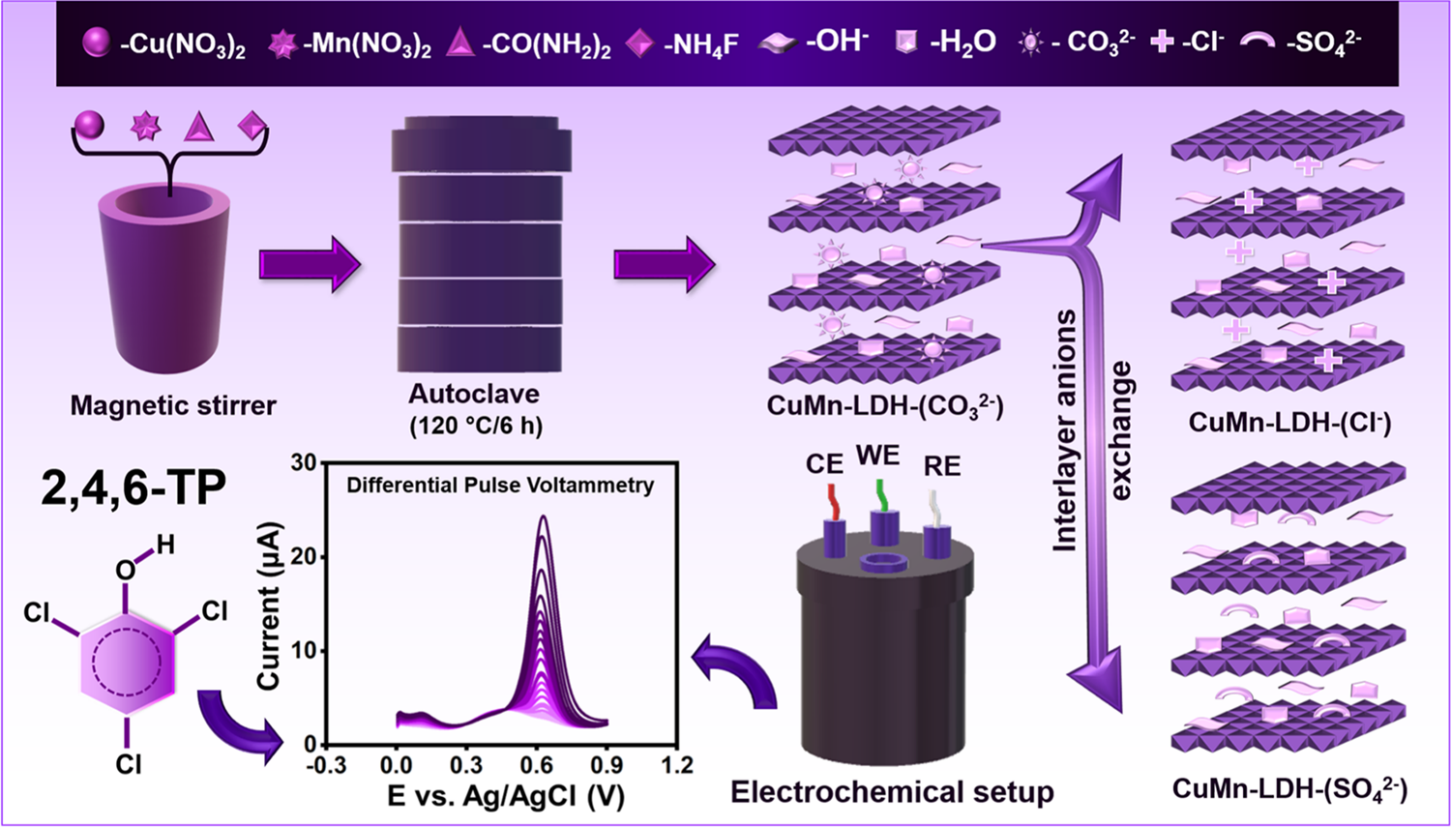
Illustration of Synthesis of Carbonated CuMn-LDH, Interlayer Anions Exchange Processes toward the TP Determination

Cl^–^ and SO_4_^2–^ intercalants are introduced into the interlayer region of CuMn-LDH by deploying an anion exchange treatment. For this purpose, 10 mg of CuMn-LDH-(CO_3_^2–^) is dissolved in 2 mL (1.0 M) of aqueous solution of KCl and vortexed for 24 h to yield Cl^–^ intercalated CuMn- LDH-(Cl^–^). Similarly, SO_4_^2–^ anions are introduced into the gallery region by dissolving 10 mg of CuMn-LDH-(CO_3_^2–^) in 2 mL of (1.0 M) aqueous solution of Na_2_SO_4_ and vortexing for 24 h. The obtained CuMn-LDH-(Cl^–^) and CuMn-LDH-(SO_4_^2–^) powders are washed and vacuum-dried before further experiments^29^ (Scheme 1).

### Preparation of the Working Electrode

A typical electrochemical sensor records the change in the current response as a function of the redox reaction of an analyte. LDH-based electrochemical sensors are often fabricated by coating a thin film of LDHs onto the working electrode surface. Prior to electrode modification, the surface of the screen-printed carbon electrode (SPCE) is cleaned using ethanol and then rinsed with DI water. A homogeneous mixture of CuMn–LDHs in DI water is prepared under vigorous stirring, cast on thoroughly cleaned SPCE, and dried to fabricate CuMn-LDHs/SPCE.

### Fish Sample Preparation

Fish samples were purchased from a local market in Taiwan. First, 6 g of fish samples were weighed, and then, using an HY-4 homogenizer set to 5000 rpm for 1 h, they were homogenized in 0.1 mol L^–1^ of EDTA and 2.0 mL of pH 7.0 McIlvaine buffer solution. After the sample was transferred into a polypropylene tube, it was mixed rapidly with 3 mL of acetonitrile and centrifuged at 5000 rpm for 10 min. Real samples were spiked with known TP concentrations and placed in 10 mL volumetric flasks at 4 °C.

## Results and Discussion

### Physical Characterization of CuMn-LDHs

**Figure 1:**
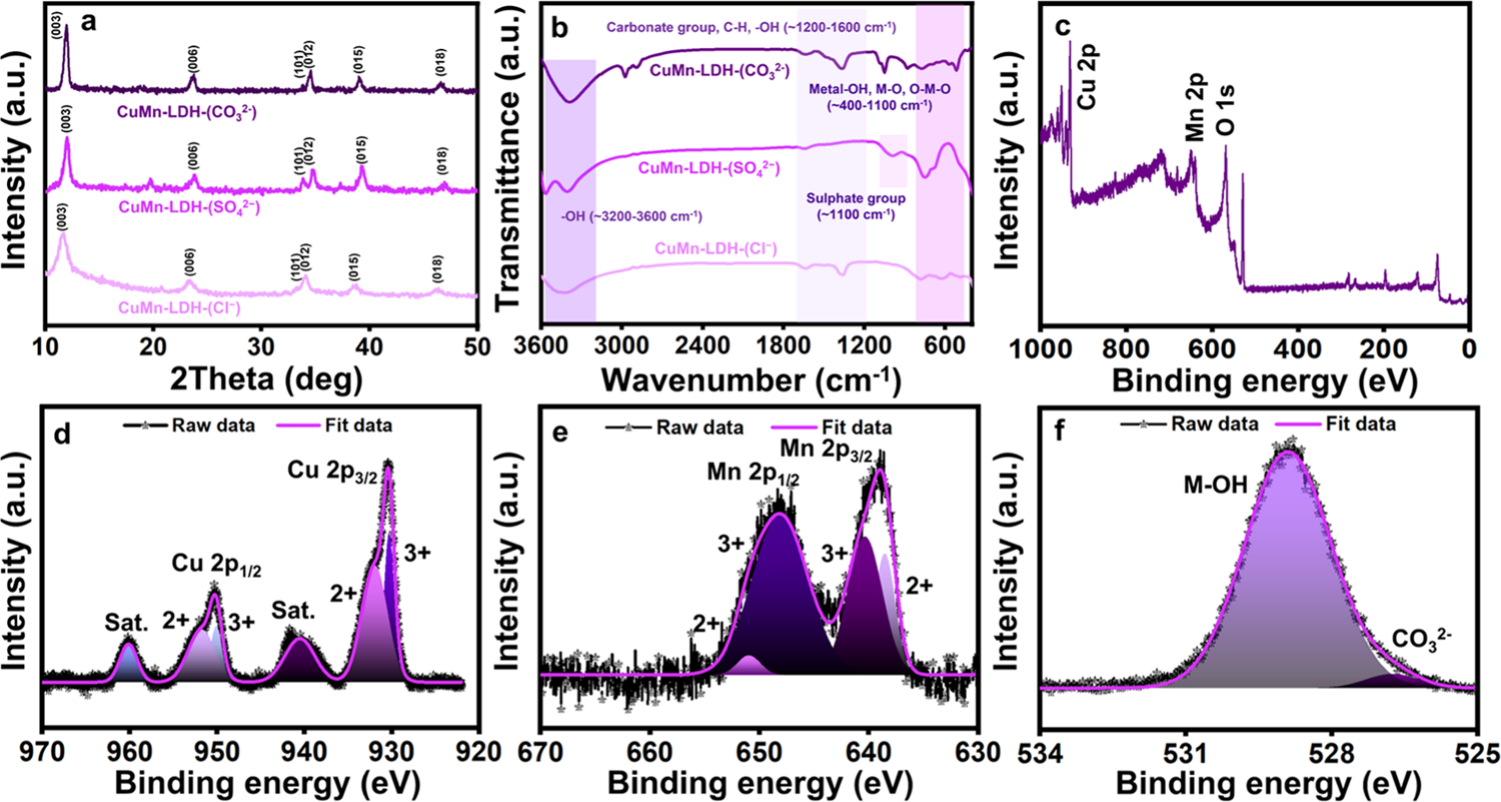
(a) XRD patterns and (b) FTIR spectra of as-prepared CuMn-LDHs. (c) XPS overall spectrum and high-resolution spectra of (d) Cu 2p, (e) Mn 2p, and (f) O 1s.

X-ray diffraction (XRD) investigations compile information concerning the crystalline phase purity, basal spacings, and crystallite sizes of the samples under analysis. XRD data of the pristine CuMn-LDHs with different interlayer anions are listed in Figure 1a.^24,31−34^ From the diffraction pattern, the 2θ values at 11.9, 23.6, 33.8, 34.5, 39.1, and 46.6° are respectively assigned to (003), (006), (101), (012), (015), and (018) crystal planes of CuMn-LDH (JCPDS no. 22–0452). These stacking planes indexed to the hexagonal lattice of the rhombohedral 3R system (*R*3*m*) authenticate the successful formation of LDH.^32^ A slight shift is observed in the position of the (003) reflection as the intercalated anions are changed from CO_3_^2–^(11.95°) to SO_4_^2–^(11.98°) or Cl^–^(11.68°).^33,34^ The changes observed with variation in the interlamellar anions can be correlated to the size and charge effects of the anions present in the gallery region and approve the successful anion exchange process.^24,31^

The FTIR spectra of CuMn–LDHs (Figure 1b)^30,35−42^ with different interlamellar anions demonstrate certain signature vibrations pertaining to the anionic species involved. For all of the prepared samples, the interlamellar hydroxyl groups of the water molecules are represented by bands centered around ∼3600 cm^–1^. The weak band focused at ∼1658 cm^–1^ is attributed to the bending vibrations arising from water molecules. The vibration bands in the 800–1700 cm^–1^ range belong to various gallery species.^35^ For CuMn-LDH-(CO_3_^2–^), the bands appearing at 1200–1500 cm^–1^ arise from the antisymmetric stretching vibrations (ν_3_) of CO_3_^2–^ anions, while the bands corresponding to the nonplanar (ν_2_) and angular (ν_4_) bending modes appear at 870 and 680 cm^–1^, respectively.^36^ Interestingly, the vibrations bands related to CO_3_^2–^ anions also make their appearance in the IR spectrum of CuMn-LDH-(SO_4_^2–^) and CuMn-LDH-(Cl^–^).^37^ This relates to the existence of CO_3_^2–^ impurities in the precursor salts, which are not entirely eliminated considering their high affinity to the LDH layers.^30,38^ The intensity of these IR impressions is weaker than the CO_3_^2–^ band for CuMn-LDH-(CO_3_^2–^), making them effortlessly distinguishable.^39^ However, the appearance of a weak band in the 3000 cm^–1^ region originating from the interaction between O–H groups and CO_3_^2–^ ions is not observed in the FTIR spectra of other anion-intercalated LDHs. The absence of this peak in the FTIR spectra of the CuMn-LDH-(SO_4_^2–^) and CuMn-LDH-(Cl^–^) is yet another confirmation for the preparation of the pure LDHs without carbonate impurity.^35^ As in the case of earlier literature studies, there are no peaks in CuMn-LDH-(Cl^–^) caused by Cl^–^ ions.^40^ For CuMn-LDH-(SO_4_^2–^), a single intense signature stretching absorption (ν_3_) associated with SO_4_^2–^ ions emerge at 1100 cm^–1^^41^ Moreover, the bands at 619 and 989 cm^–1^ are connected to the bending (ν_2_) and symmetric stretching (ν_1_) modes of SO_4_^2–^ ions, respectively.^41,42^

In order to obtain a comprehensive understanding of the chemical composition and phases of CuMn-LDH, we acquired XPS spectra were acquired. The XPS survey spectrum of CuMn-LDH is depicted in Figure 1c; it reveals the existence of Cu 2p, Mn 2p, O 1s, and C 1s elements. Two main peaks corresponding to Cu 2p3/2 at 930.38 eV (3+) and 931.99 eV (2+) and Cu 2p1/2 at 950.06 eV (3+) and 951.76 eV (2+), respectively, are depicted in Figure 1d, which illustrates high-resolution Cu 2p XPS spectra. The 960.18 and 940.01 eV binding energy values, respectively, correspond to the satellite peaks of Cu 2p3/2 and Cu 2p1/2. In the same way, the spectrum of Mn 2p illustrated in Figure 1e reveals the presence of two distinct Mn species: Mn^3+^ 2p3/2 and Mn^2+^ 2p3/2 are indicated by the fitting peaks at 640.33 and 638.49 eV, respectively, and Mn^3+^ 2p1/2 and Mn^2+^ 2p1/2 are designated by the peaks at 648.25 and 651.06 eV, respectively. The prominent peak observed at 528.90 eV in the high-resolution O 1s spectrum (Figure 1f) corresponds to the lattice oxygen atoms present in the CuMn-LDH. Additionally, the peak at 526.68 eV is indicative of the interlayer carbonate atoms in the CuMn-LDH. The peak observed at 282.13 and 286.66 eV in the high-resolution C 1s spectrum is indicative of the interlayer carbonate atoms in the CuMn-LDH present in the CuMn-LDH (Figure S1). In complete agreement with the EDX, the XPS result confirms the formation of CuMn–LDH without revealing any impurities. In complete agreement with the EDX, the XPS result confirms the formation of CuMn–LDH without revealing any impurities.

**Figure 2:**
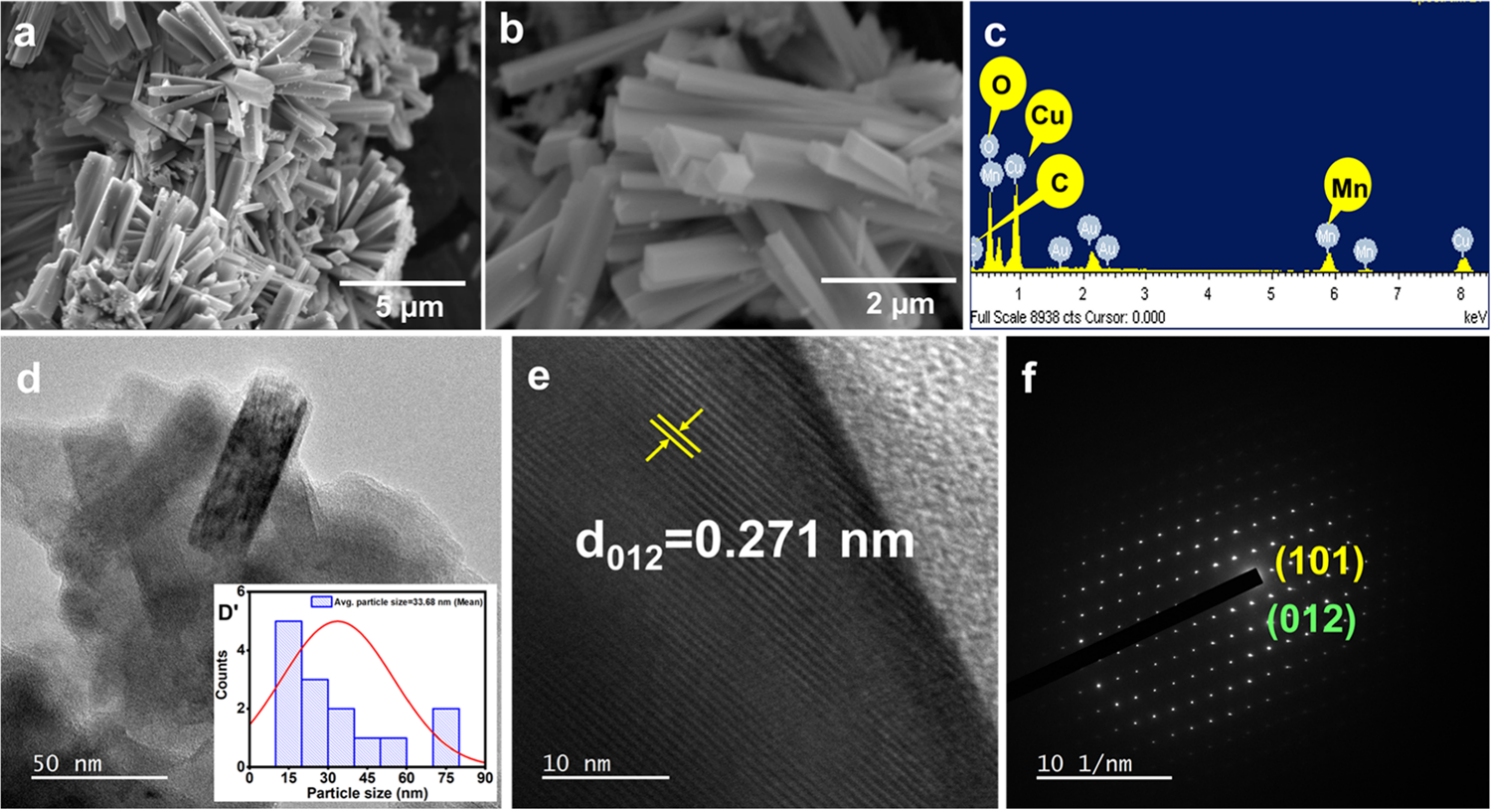
(a, b) SEM images and (c) EDX spectrum of CuMn-LDH. (d, e) HRTEM images and (f) SAED patterns of CuMn-LDH. (D’) CuMn-LDH size distribution plot.

The microstructural specifics of the developed CuMn-LDHs are explored with the assistance of scanning electron microscopy (SEM) and transmission electron microscopy (TEM). From Figure 2a,b, CuMn-LDHs are found to inherit a rod-like structure typical of LDH materials. Controlling nucleation and development during coprecipitation is highly difficult, and thus, most often LDHs crystallize as sheets. However, due to the inherent constraints of multilayer stacking, active center exposure is severely limited, and conductivity declines. Technological advancements now allow for the development of LDHs that have classified morphologies, increased specific surface areas, and fast electron transit kinetics. The use of urea-assisted hydrothermal synthesis and typical conventional methods has been reportedly useful in producing LDH with rod-like morphology.^43,44^ In Figures 2c and S2a,b, the EDX profile reveals exclusive signals corresponding to the presence of constituent elements such as Cu (∼45.53%), Mn (∼7.73%), C (∼4.12%), S (∼6.49%), Cl (∼3.44%) and O (∼34.49%) with a uniform elemental distribution. The high-resolution TEM images (Figure 2d) confirm the rod structure of the prepared LDHs. The high-resolution TEM images (Figure 2d,D’) confirm the rod structure of the prepared LDHs supported with the SEM images. The lattice spacing of the prepared CuMn-LDH-(CO_3_^2–^) sample is 0.271 nm and is in accordance with the XRD plane of (012) (Figure 2e). The results of the electron microscopy examination for CuMn-LDHs intercalated with various anions reveal that the anion exchange process takes place without disrupting the microstructure and surface attributes of the LDH structures. According to the SAED patterns (Figure 2f), CuMn-LDH has a crystalline structure, as depicted by the appearance of bright spots.

### Electrochemical Behavior of CuMn–LDHs

**Figure 3:**
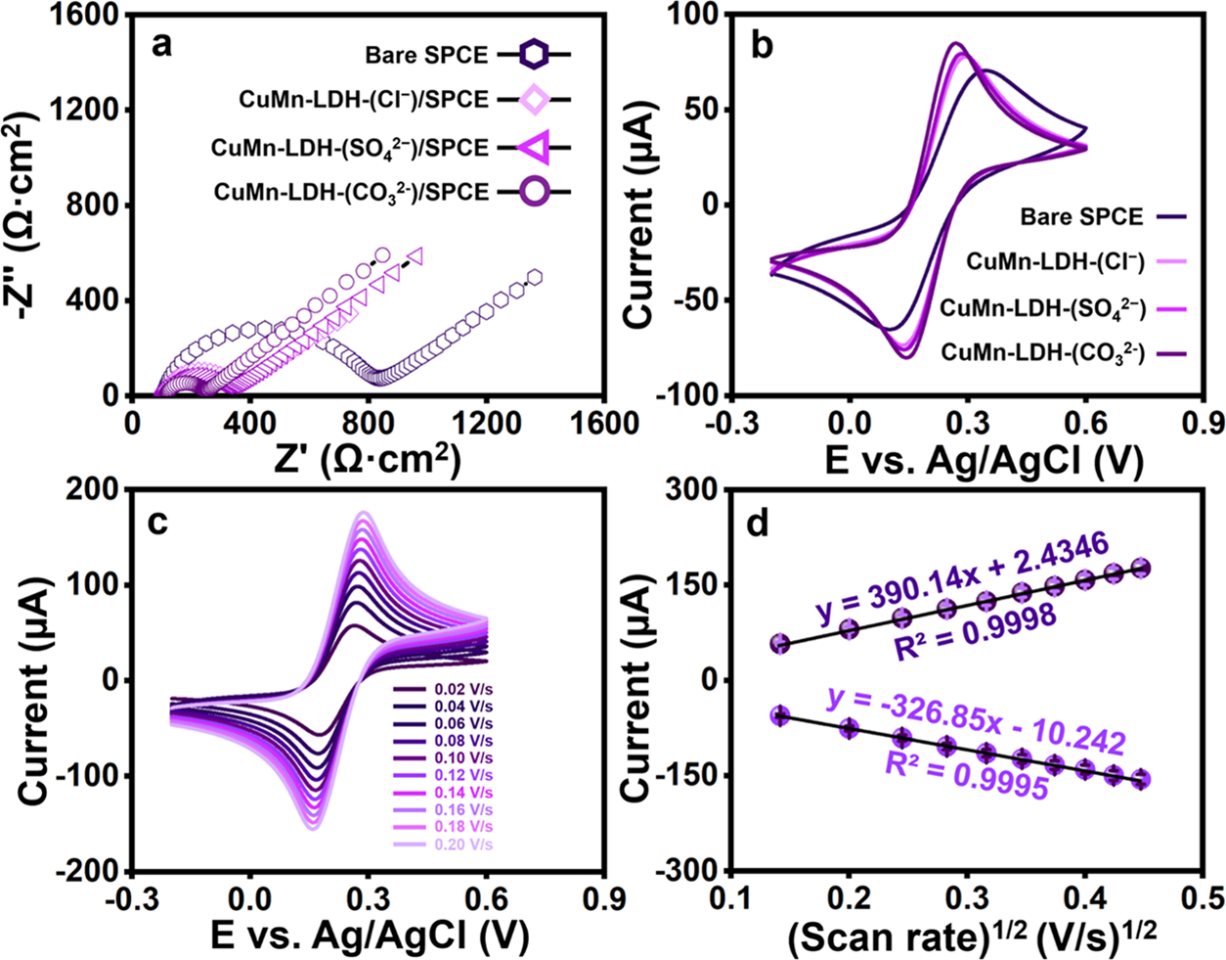
(a) EIS and (b) CVs of bare–, CuMn–LDH-(Cl^–^)–, CuMn–LDH-(SO_4_^2–^)–, and CuMn–LDH-(CO_3_^2–^) modified SPCEs in K_4_[Fe(CN)_6_]/K_3_[Fe(CN)_6_]. (c) Different scan rates (0.02–0.20 V·s^–1^) of CuMn–LDH-(CO_3_^2–^) in K_4_[Fe(CN)_6_]/K_3_[Fe(CN)_6_] and (d) corresponding calibration plot of scan rate (V·s^–1^)^1/2^ vs current (μA).

Electron impedance spectroscopy (EIS) is employed to comprehensively evaluate the influence of intercalated anions on the interfacial characteristics of CuMn–LDH-coated SPCE. The EIS analysis is carried out in a [Fe(CN)_6_]^3–/4–^ redox probe. Using Randle’s equivalent circuit, the EIS data is fitted with *R*_ct_, *R*_s_, *Z*_w_, and *C*_dl_, respectively, representing charge transfer resistance, solution resistance, Warburg impedance, and double-layer electron-transfer resistance.^45^ The Nyquist plots given in Figure 3a correlate the diameter of the semicircular arc to the electron-transfer resistance. Materials with low *R*_*c*t_ values are generally ideal for initiating effective charge transfer. Due to the high resistance presented by bare SPCE (*R*_ct_ = 710.23 Ω·cm^2^), surface treatment with the proper electrocatalysts essentially improves the transport properties at the electrode. The EIS analysis of different CuMn control electrodes confirms that anion intercalation results in varying degrees of resistance to the overall ion transit. The efficiency of transport kinetics at the CuMn-LDH-(SO_4_^2–^)/SPCE and CuMn-LDH(Cl^–^)/SPCE are comparatively lower, denoted by their *R*_ct_ values (217.38 and 255.09 Ω·cm^2^). In comparison, CuMn-LDH-(CO_3_^2–^)/SPCE facilitates a smooth ion transfer, as inferred from the minimal *R*_ct_ value (134.52 Ω·cm^2^). Thus, CuMn-LDH-(CO_3_^2–^)/SPCE offers more fluid carrier movement, improving electrochemical activity.

Further, the cyclic voltammograms (CV) of bare SPCE, CuMn-LDH-(CO_3_^2–^)/SPCE, CuMn-LDH-(SO_4_^2–^)/SPCE, and CuMn-LDH-(Cl^–^)/SPCE obtained in a solution containing 5.0 mM [Fe(CN)_6_]^3–/4–^ and 0.1 M KCl are displayed in Figure 3b. On account of the quasi-reversible nature of the [Fe(CN)_6_]^3–/4–^ redox couple, all modified electrodes exhibit significant redox peaks. CuMn-LDH-(CO_3_^2–^)/SPCE exhibits an augmented current responsiveness relative to those of other modified electrodes. Further, the peak-to-peak separation (Δ*E*_p_) values of the bare SPCE, CuMn-LDH-(CO_3_^2–^)/SPCE, CuMn-LDH-(SO_4_^2–^)/SPCE and CuMn-LDH-(Cl^–^)/SPCE are found to be 230, 120, 152, 155 mV (Table 1). The obtained CV results reveal that the electrochemical reaction is quite sensitive to the characteristic nature of gallery anions. Figure 3c displays the voltammogram profile of CuMn-LDH-(CO_3_^2–^)/SPCE by increasing the scan rate from 0.02 to 0.2 V s^–1^ in 0.1 M KCl containing 5.0 mM [Fe(CN)_6_]^3–/4–^. As the scan rate is steadily increased, the peak potential appears to increase gradually. The associated calibration plot is shown in Figure 3d.

**Table 1:** Analytical Results from Electrochemical Measurements on the Redox Probe ([Fe(CN)_6_]^3–/4^)

electrodes	EIS (Ω)	*I*_pa_ (μA)	*E*_pa_ (V)	*I*_pc_ (μA)	*E*_pc_ (V)	Δ*E*_pa_ – *E*_pc_ (V)
bare	710.23	70.91	0.34	–66.38	0.11	0.23
CuMn-LDH-(Cl^–^)	255.09	76.40	0.29	–72.89	0.135	0.155
CuMn-LDH-(SO_4_^2–^)	217.38	79.62	0.28	–76.40	0.138	0.152
CuMn-LDH-(CO_3_^2–^)	134.52	85.39	0.26	–80.13	0.14	0.12

As the electrode responses greatly depend on the surface area available on the working electrode for catalyzing electrochemical reactions, the electrochemically active surface area (A) calculations for various modified SPCEs are performed with the use of Randles-Sevcik Equation^46,47^ (eq 6).
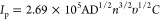
6

The various parameters i.e., *I*_p_, *D*, *n*, *v*, and *C* used in the above equation respectively represent the peak current response (μA), diffusion coefficient (7.6 × 10^–6^ cm^2^ s^–1^), number of electrons participating in the redox conversion (*n* = 1), scan rate (V s^–1^), and bulk concentration of [Fe(CN)_6_]^3–/4–^. The electrochemical active surface area of CuMn-LDH-(CO_3_^2–^)/SPCE (0.105 cm^2^) is enhanced, enabling faster diffusion of ions across exposed active sites. The superior surface characteristics of CuMn-LDH-(CO_3_^2–^)/SPCE are in good agreement with those of the EIS and CV profiles. As a result of faster diffusion of ions across exposed active sites, efficiency is indeed enhanced for CuMn-LDH-(CO_3_^2–^)/SPCE. It follows that electrocatalysis activity is not determined by basal spacing.^30^ Having analyzed the above findings, it is evident that intercalated CO_3_^2–^ anions significantly enhance the electrochemical performance of CuMn–LDHs and are thus used for further electrochemical studies.

**Figure 4:**
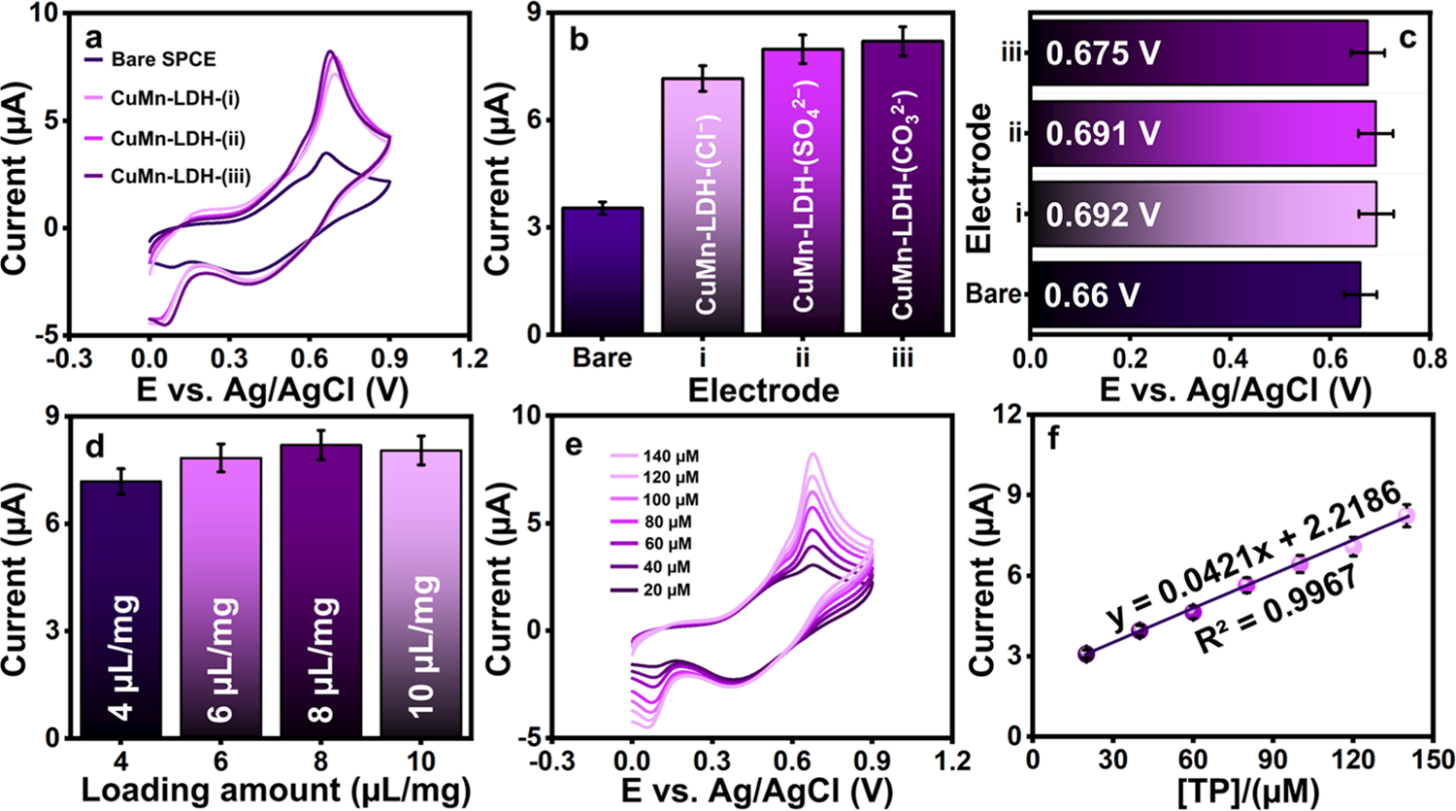
(a) CV films of bare and modified electrodes such as CuMn-LDH-(CO_3_^2–^)/SPCE, CuMn-LDH-(SO_4_^2–^)/SPCE, and CuMn-LDH(Cl^–^)/SPCE in the presence of TP and its bar diagram for obtained TP (b) peak currents (μA) and (c) potential (V) versus electrodes. (d) Effect of different modifying amounts of CuMn-LDH-(CO_3_^2–^) to electrode. (e) CV response of different concentrations of TP (20–140 μM) in pH 7 at CuMn-LDH-(CO_3_^2–^) and (f) calibration plot of concentration (μM) vs current (μA).

To investigate the behavior of various CuMn-LDHs/SPCE toward the detection of TP, cyclic voltammetric (CV) analysis is employed. Briefly, the voltammogram profiles for the bare and LDH-modified SPCEs recorded using a 0.1 M PB (pH 7) containing TP at a scan rate of 50 mVs^–1^ within a potential range of from 0.0 to 0.9 V are shown in Figure 4a. As a consequence of the inherently low ionic conductivity of the unmodified SPCE surface, the bare SPCE responds poorly toward TP. The exceptional current response is witnessed for CuMn-LDH-(CO_3_^2–^)/SPCE (*I*_pa_ = 8.2 μA at the potential *E*_pa_ = 0.675 V) compared to bare SPCE (*I*_pa_= 3.54 μA at the potential *E*_pa_ = 0.66 V), CuMn-LDH-(SO_4_^2–^)/SPCE (*I*_pa_= 7.98 μA at the potential *E*_pa_ = 0.691 V) and CuMn-LDH-(Cl^–^)/SPCE (*I*_pa_= 7.16 μA at the potential *E*_pa_ = 0.692 V). Figure 4b,c show the corresponding bar diagrams. Hence, it is further confirmed that the intercalation of CO_3_^2–^ leads to an increased electrical conductivity of CuMn-LDH toward the detection of TP.

### Optimization of Detection Parameters

For efficient electrode composition and analysis conditions, the optimization of reaction parameters such as loading amount, concentration, scan rate, and pH is indispensable. Figure 4e shows current responses obtained for varied surface coverages from 4 to 10 mg^–1^ μL. The oxidation current response increases from 4 to 8 mg^–1^ μL but decreases at 10 mg^–1^ μL. This depends on the coating thickness and passivation. Thin coatings have poor target analyte adsorption, which leads to poor signal amplification at low current values. As the CuMn-LDH-(CO_3_^2–^) volume increases, modifier film thickness on SPCE increases, allowing solute penetration to the active core and stimulating the dynamic interaction of the developed electrode to TP molecules to improve current responses. As the coating thickness increases, the electrode saturation blocks ions from crossing the printed carbon layer and coated CuMn-LDH-(CO_3_^2–^) layer, reducing TP signals. Thus, all the other electrochemical experiments were performed by utilizing 8 μL mg^–1^ of CuMn-LDH-(CO_3_^2–^) with TP. Further, Figure 4e illustrates the CV responses for CuMn-LDH-(CO_3_^2–^)/SPCE obtained by varying the concentrations of TP in 0.1 M PB at pH 7.0 with a scan rate of 50 m V s^–1^. A sharp anodic response is observed corresponding to the oxidation of TP at a potential value of ∼0.67 V, which gradually increases as the concentration of TP is increased from 20 to 140 μM. The corresponding calibration plot given in Figure 4f presents a linear regression equation and correlation coefficient of (eq 7)
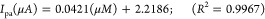
7

**Figure 5:**
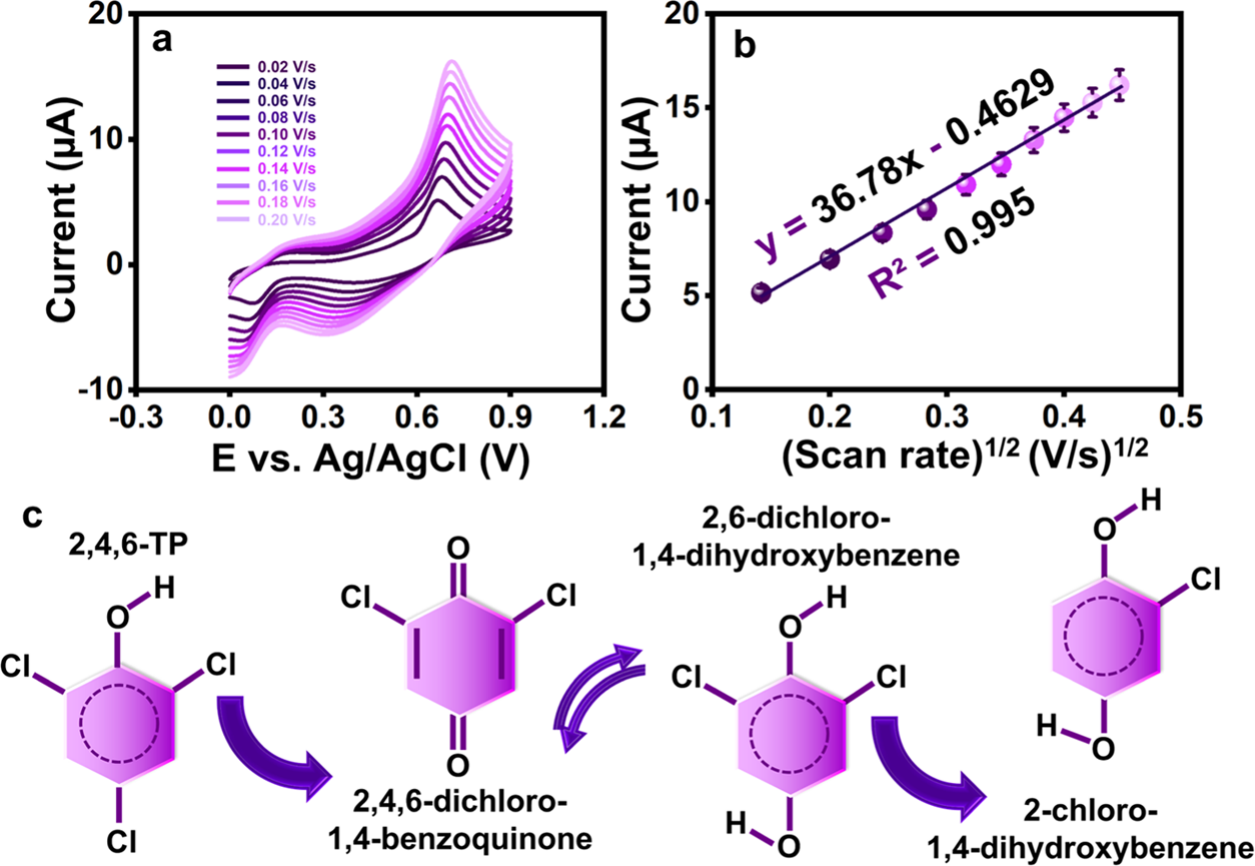
(a) Different scan rates (0.02–0.20 V s^–1^) in the presence of TP and (b) linear plot of scan rate (V s^–1^)^1/2^ vs current (μA) of TP. (c) TP has a possible electro-oxidation mechanism.

The effect of the scan rate on TP oxidation for CuMn-LDH-(CO_3_^2–^) in 0.1 M PB (pH 7) containing TP at various scan rates (0.02–0.20 V s^–1^) is demonstrated in Figure 5a. The CV response shows a significant increase in peak currents due to the oxidation of TP, with scanning rates increasing from 0.02–0.20 V s^–1^. Kinetic restriction and mass transfer may have caused the peak potentials for TP oxidation to shift gradually to the positive side. Figure 5b shows the linear relationship between the anodic peak currents and the square root of scan rates (*I*_pa_ (μA) = −36.78 (V s^–1^) – 0.4629; *R*^2^ = 0.995) suggests a diffusion-controlled electrochemical process for the electron transfer at CuMn-LDH-(CO_3_^2–^)/SPCE. The mechanism for the electrochemical oxidation of TP is provided in Figure 5c. The electrochemical mechanism of 2,4,6-trichlorophenol (TP) follows a typical pattern of phenol derivatives. In the initial step, the hydroxyl group of TP is oxidized, and the H^+^ ion is released into the solution. The obtained unstable moiety immediately undergoes the nucleophilic substitution and produces 2,5-dichloro-1,4-benzoquinone by eliminating the chlorine at the para position and producing a small amount of HCl as the addition product. The chlorine atom in para- position is the probable candidate due to less steric hindrance. The irreversible oxidation of the hydroxyl group is indicated by the presence of a strong peak at ∼0.69 V, where the maximum current response corresponding to TP electro-oxidation was obtained upon using the CuMn-LDH-(CO_3_^2–^). The reversible pair of peaks at lower potential is ascribed to the formation of quinones. Overall, the electro-oxidation of TP involves a transfer of two protons and two electrons.^20,48−50^

By performing CV studies at different pH levels, we analyzed the effect of pH on the electrochemical behavior of the CuMn-LDH-(CO_3_^2–^) /SPCE system is analyzed. The cyclic voltammetric responses of CuMn-LDH-(CO_3_^2–^)/SPCE toward TP at a scan rate of 50 mV s^–1^ are presented in Figure S3. An increase in current response from pH 3 to 7 and the subsequent decrease in anodic peak current (pH 7–11) helps to confirm pH 7 as the optimal reaction pH. Additionally, the progressive linear shift toward the negative potential was observed, marking the involvement of protons in the electro-oxidation mechanism of TP on CuMn-LDH-(CO_3_^2–^)/SPCE. Accordingly, all electrochemical experiments were further performed in 0.1 M pH 7.0 with TP.

### Electrochemical Determination of TP by Differential Pulse Voltammetry (DPV) Analysis

**Figure 6:**
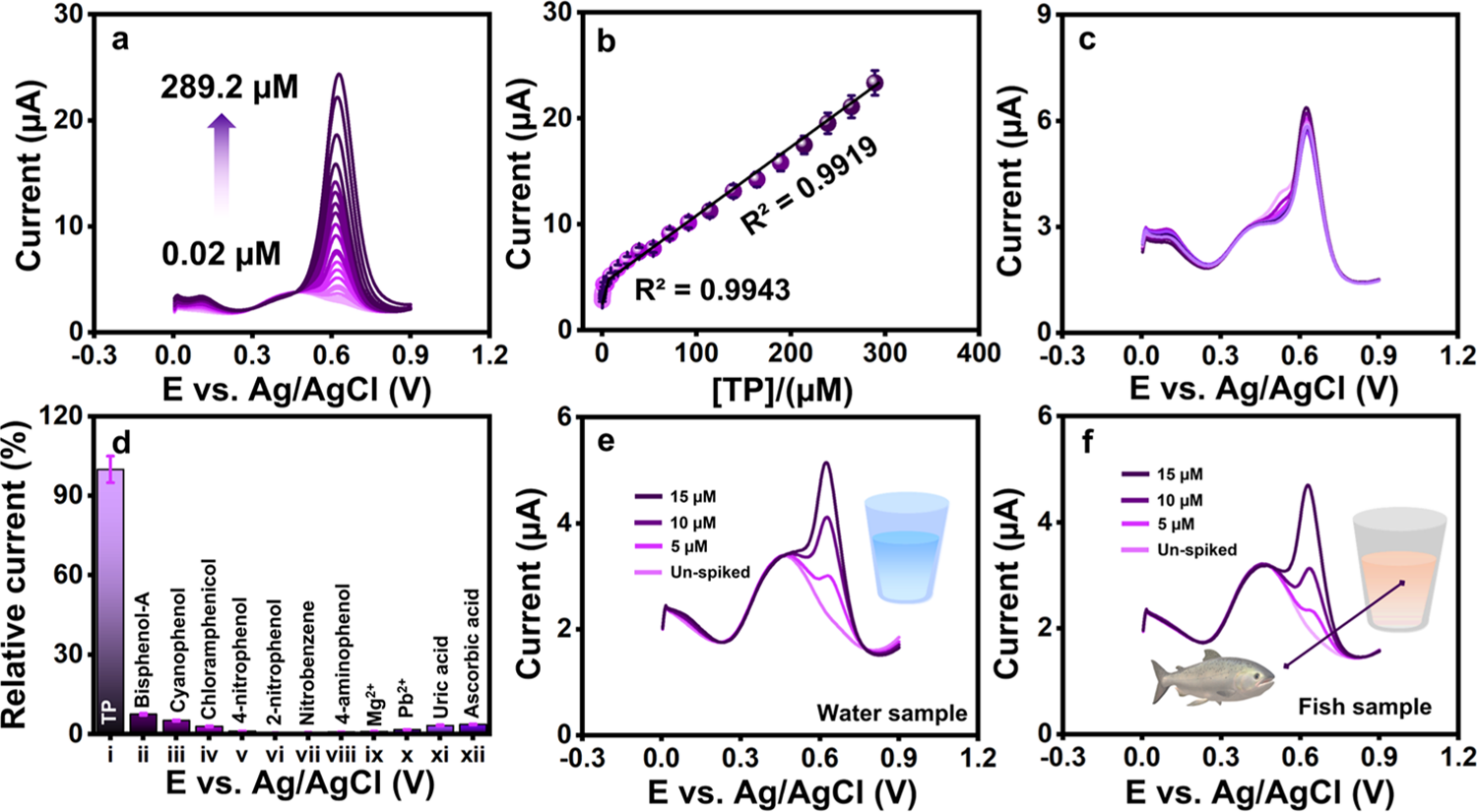
(a) DPV of CuMn-LDH-(CO_3_^2–^)/SPCE in various concentrations (0.02–289.2 μM) of TP at 0.1 M (PB) and (b) calibration plot for TP peak current (μA) versus TP concentration (μM). **(**c, d**)** Anti-interference ability of CuMn-LDH-(CO_3_^2–^)/SPCE in the presence of TP and co-presence of other interfering compounds. (e, f) DPV of CuMn-LDH-(CO_3_^2–^)/SPCE real-time analysis in water and fish samples spiked of TP.

DPV method offers an efficient sensing strategy with high sensitivity and resolution for trace-level analyte detection and hence is chosen for the electrochemical quantification of TP. DPV response profile of CuMn-LDH-(CO_3_^2–^)/SPCE toward the concurrent increase in TP concentration under optimized conditions in 0.1 M PB is recorded in Figure 6a. The conditions set for DPV studies are as follows: The DPV parameters: initial 0 to 0.9 V applied potential window; In cr *E*(*V*): 0.004; 0.05 V for the pulse amplitude; 0.025 s for the pulse width (sec); 0.0167 s for the sampling width; 0.5 s for the pulse period; 2 s for the quiet time; 1.e^–004^ for the sensitivity (A/V). On altering the concentration of TP (0.02–289.2 μM), an escalation is observed in the oxidation peak current response. The calibration curve constructed corresponding to these changes in Figure 6b reveals a wide linear range of 0.02–289.2 μM. Using the IUPAC 3σ method (S/N = 3),^51−53^ the limit of detection (LOD) is calculated to be 0.0026 μM. A comprehensive performance comparison of CuMn-LDH-(CO_3_^2–^)/SPCE with other reported analytical systems listed in Table 2 confirms the superiority of the as-prepared sensor.

**Table 2:** Comparison of Detection Limit of the Developed CuMn-LDH Electrode

**electrodes**	**linear range (**μM**)**	**LOD (**μM**)**	**real samples**	**refs**
MIP/Mn/ZnS/QD	0.1–30	0.035	water	(1)
GO@SrTiO_3_	0.01–434.4	0.0032	water	(3)
CNTs–OH/PtNPs/RhB	5.0–175.0	1.55	water	(4)
CS@Ag@GO	0.03–35	0.0097	water	(5)
CuO/Nafion	1–120	0.046	water	(8)
Ag_2_O NPs/GCE	0.005–1261.6	0.0027	water	(18)
MnV_2_O_6_/SPCE	0.01–1340	0.009	-	(20)
**CuMn-LDH**	**0.02**–**289.2**	**0.0026**	**water, fish**	**this work**

### Selectivity, Cyclic Stability, and Reproducibility Studies

Optimal electrochemical sensing efficiency demands a highly selective catalyst material that responds only to the target analyte. Consequently, the selectivity of CuMn-LDH-(CO_3_^2–^)/SPCE is investigated by analyzing the performance of TP in the presence of different foreign interfering agents (Figure 6c). Interferons such as bisphenol A, cyanophenol, chloramphenicol, 4-nitrophenol, 2-nitrophenol, nitrobenzene, 4-aminophenol, Mg^2+^, Pb^2+^, uric acid, and ascorbic acid were added in a 5-fold excess concentration compared to TP. The corresponding bar diagram in Figure 6d reveals that the presence of interferons has a negligible impact on the selectivity of CuMn-LDH-(CO_3_^2–^)/SPCE toward TP. The sensing reproducibility of the CuMn-LDH-(CO_3_^2–^)/SPCE sensors toward TP is evaluated by analyzing the DPV performance of five independent electrodes under similar experimental settings. The results are presented in Figure S4. A relative standard deviation (RSD) of ±2.54% confirms the proposed sensor’s reproducible maintainability. In order to evaluate the stability of the sensor, its DPV performance for the electrochemical detection of TP was monitored for up to 20 days (Figure S5). The CuMn-LDH-(CO_3_^2–^)/SPCE displayed a highly steady current output for TP without any significant drop even after 20 days. Less than 10% of its initial current response was retained by the electrode, indicating its high durability.

### Real Sample Analysis

An evaluation of the applicability of the sensor described is being conducted by using real-world samples. DPV analysis is performed on river water and fish samples to determine whether the CuMn-LDH-(CO_3_^2–^) modified sensor is practicable (Figure 6e,f). Fish samples were sourced from a local market in Xindian City, Taiwan, and the river water was obtained from the Xindian River (a section of Taipei). Real samples, including river water and fish, were alternatives to the experiment. No voltammetry response suggests the absence of TP in these unspiked samples. So, we utilized a standard additive approach to preparing the TP concentration. The TP concentration is determined using the standard addition method, and the recovery rates are indicated in Table 3. Based on these results, CuMn-LDH-(CO_3_^2–^)/SPCE can be a valuable tool for monitoring TP in real-world samples since their recovery ranges are within acceptable limits.

**Table 3:** Detection of TP in Different Real Samples Using CuMn-LDH-(CO_3_^2–^)/SPCE in the Presence of Different Additions of TP. (*n* = 3)

		**detected (μM)**		**detection rate (%)**
**samples**	**added (μM)**	**DPV**	**HPLC**	(mean ± RSD)(*n* = 3)
river water	0			
	5	4.88	4.95	97.60 ± 0.019
	10	9.91	9.98	99.10 ± 0.04
	15	14.79	75.06	98.60 ± 0.013
fish	0			
	5	4.93	4.97	98.60 ± 0.011
	10	9.87	9.99	98.70 ± 0.002
	15	14.89	14.92	99.26 ± 0.07

## Conclusions

Chlorophenols (CPs), a class of persistent organic pollutants, have garnered considerable attention because of the substantial risks that they represent to the environment and public health. Recently, there have been concerns about the effectiveness of their monitoring. In an attempt to enhance the detection capability of LDH-based electrochemical sensors for TP detection, we fabricated a series of CuMn-LDHs by using a hydrothermal approach. The intercalants were varied by anion exchange and were compared to analyze the effect of anions on the electrochemical capability of the prepared LDH. The findings revealed significant enrichment in the electrocatalytic oxidation ability of the CuMn-LDH toward TP molecules with the incorporation of CO_3_^2–^ anion into the gallery region, implying that the electro-oxidation of TP is a function of anion basicity. Under the optimum conditions, the electrochemical sensor based on CuMn-LDH-(CO_3_^2–^) performs admirably for TP determination. Featuring outstanding stability, selectivity, and flexibility, the proposed electrode offers considerable potential for real-time sensing applications.

## Supporting Information Available

Instrumentation and methods; XPS high-resolution spectrum of C 1s; EDX spectra of CuMn-LDH-(SO_4_^2–^) and CuMn-LDH-(Cl^–^); effect of supporting various electrolytes; reproducibility measurement of CuMn(CO_3_^2–^)-LDH/SPCE; storage stability of CuMn-LDH with the presence of TP (PDF)
