# Supplementary material for: Two-Dimensional CuMn-Layered Double Hydroxides: A Study of Interlayer Anion Variants on the Electrochemical Sensing of Trichlorophenol
Source: Inorg Chem. 2024 Jan 23;63(5):2833–43. doi: 10.1021/acs.inorgchem.3c04568 (PMC10848258; doi:10.1021/acs.inorgchem.3c04568)
Supplement: Supplementary file 1 — ic3c04568_si_001.pdf [file ic3c04568_si_001.pdf]

Supporting information

Two Dimensional CuMn-Layered Double Hydroxides: A Study of Interlayer Anion Variants on the Electrochemical Sensing of Trichlorophenol

Balasubramanian Sriram,[a,¥] Megha Maria Stanley,[¥,b] Sea-Fue Wang,[a,] Yung-Fu Hsu,[a] Mary George[b,¥]*

[a]Department of Materials and Mineral Resources Engineering, National Taipei University of Technology, Taipei 106, Taiwan.

[b]Department of Chemistry, Stella Maris College, Affiliated to the University of Madras, Chennai, Tamil Nadu 600086, India.

Corresponding author:

***Sea-Fue Wang:** sfwang@ntut.edu.tw

[¥]*Authors have contributed equally to this work.*

No. of pages: 5

No. of figures: 5

Entry	*Table of content*	*Page No.*
Instrumentation and methods		*S2*
Figure S1	XPS high-resolution spectrum of C 1s	*S3*
Figure S2	EDX spectra's of CuMn-LDH-(SO_4^{2-}) and CuMn-LDH-(Cl^-)	*S3*
Figure S3	Effect of supporting various electrolytes	*S4*
Figure S4	Reproducibility measurement of CuMn(CO_3^{2-})-LDH/SPCE	*S4*
Figure S5	Storage stability of CuMn-LDH with the presence of TP	*S5*

Instrumentation and methods

An SEM instrument (JSM-6510LV, JEOL) operated at 15 kV and 10 μA and a high resolution (HR) transmission electron microscope (TEM) (JEOL JEM-2100F (HR)) operating at 200 kV and by energy-dispersive X-ray spectroscopy using EDAX AMETEK Inc., DigitalMicrograph® software were used to investigate the morphology and chemical composition of samples. Structural characteristic such as phase configuration, Fourier transform infrared spectra utilized Perkin Elmer spectrometer and Horiba HR 800UV confocal spectrophotometer, respectively. The electrochemical characteristics were explored using electrochemical impedance spectroscopy (EIS) Autolab. Furthermore, CHI 6171D electrochemical workstation was to carry out electrochemical measurements like cyclic voltammetry (CV) and differential pulse voltammetry (DPV) in a conventional three-electrode cell. Here, the modified SPCE (surface area = 0.071 cm^2), saturated Ag|AgCl, and Pt wire are active as working, reference, and counter electrodes, respectively.

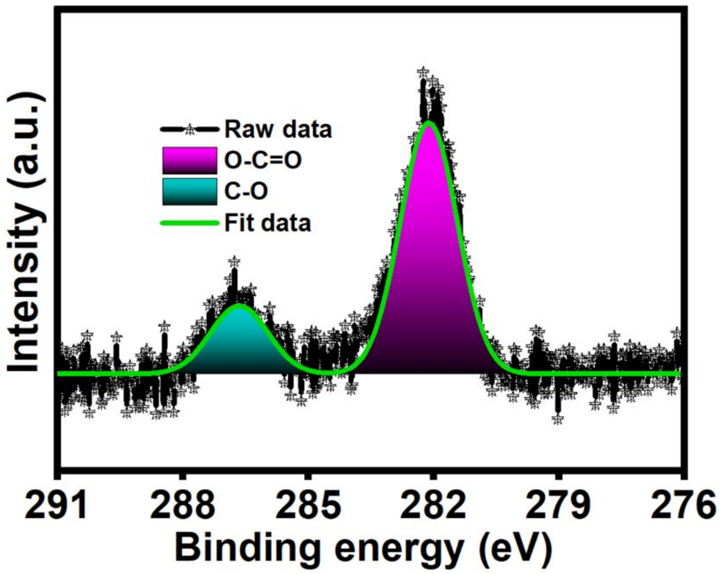

Figure S1. XPS high-resolution spectrum of C 1s.

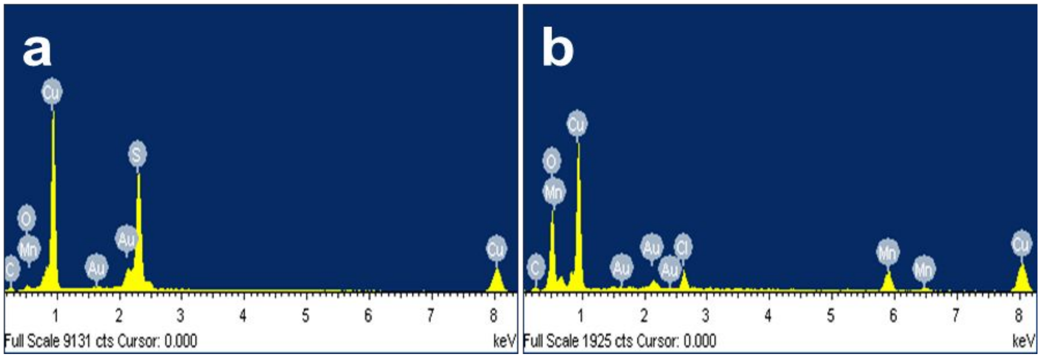

Figure S2. EDX spectra's of CuMn-LDH-(SO_4^{2-}) and CuMn-LDH-(Cl^-).

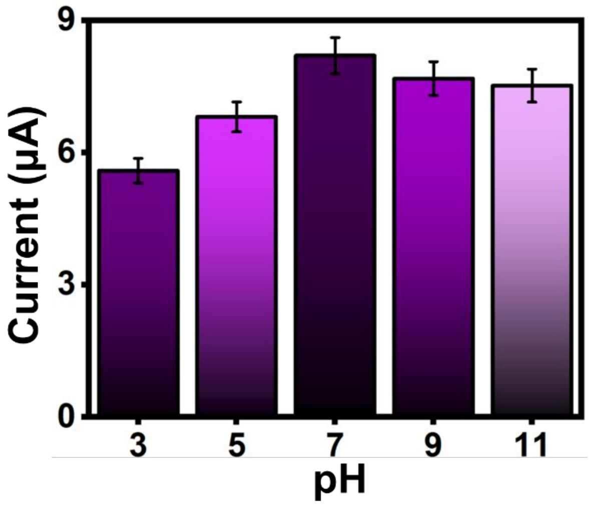

Figure S3. Effect of supporting various electrolytes

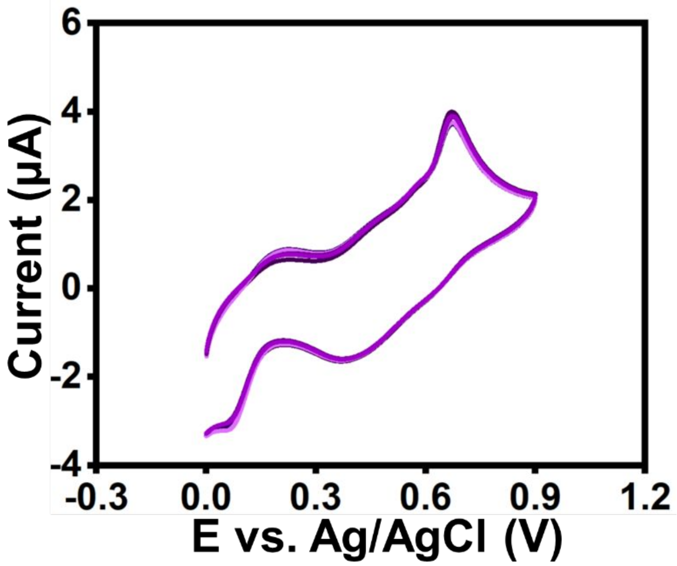

Figure S4. Reproducibility measurement of CuMn(CO_3^{2-})-LDH/SPCE in the presence of TP.

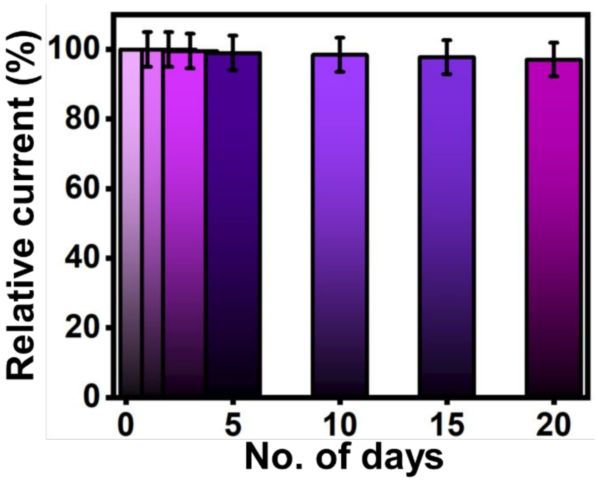

Figure S5. Storage stability of CuMn-LDH with the presence of TP.
